# Endovascular Salvage of Native Arteriovenous Fistulas in Hemodialysis Patients: Assisted Primary Patency Outcomes of a Single-Center Study

**DOI:** 10.7759/cureus.103150

**Published:** 2026-02-07

**Authors:** Oscar F Vargas, Juliana Salcedo-Mesa, Valentina Lugo-Mesa, Ivan R Nieto

**Affiliations:** 1 Interventional Radiology, Hospital Departamental de Villavicencio, Villavicencio, COL; 2 Medicine, Pontificia Universidad Javeriana, Bogotá, DC, COL; 3 Epidemiology, Universidad de los Andes, Bogotá, DC, COL; 4 Nephrology, Hospital Departamental de Villavicencio, Villavicencio, COL

**Keywords:** angioplasty, arteriovenous fistula, cross sectional study, epidemiology, hemodialysis access, interventional radiology, stent, thrombosis, vascular fistula, vascular patency

## Abstract

Introduction: Patients with end-stage kidney disease (ESKD) on hemodialysis (HD) require a functional vascular access, of which the best option is a native arteriovenous fistula (AVFn). These fistulas may fail due to multiple factors, which may delay HD, and salvage endovascular interventions are indicated to restore fistula patency. Given the increasing incidence of ESKD, it is essential to describe demographic and clinical characteristics of patients with AVFn, as well as their patency outcomes after the first salvage intervention. The purpose of this study is to evaluate the assisted primary patency at one, six, and 12 months in a third-level hospital in Colombia.

Methods: We conducted a retrospective cross-sectional study to characterize patients undergoing endovascular salvage of arteriovenous fistulas (AVFs) and describe assisted patency at one, six, and 12 months. Our study included patients treated between January 2023 and June 2025 at the Hospital Departamental de Villavicencio, a tertiary-level hospital and regional referral center in Colombia. Exclusion criteria included patients with prior open surgical salvage, undetermined procedural success, or incomplete medical records. Data were obtained from electronic medical records, which included demographic, clinical, and procedural characteristics. Descriptive statistics were performed using RStudio (RStudio, PBC, Boston, MA).

Results: A total of 43 patients were included in the study (mean age 59 years, 70% male). Hypertension was the leading cause of ESKD (38, 88%) and the most frequent comorbidity (42, 98%). Thrombosis was the main cause of AVFn dysfunction (34, 79%). Most fistulas were brachiocephalic (32, 74%) and left-sided (26, 60%). The median time from dysfunction to salvage was 8 days. Angioplasty was performed in 42 patients (98%), followed by thrombectomy in 22 (51%), stent placement in 12 (28%), and thromboaspiration in 12 (28%). Primary assisted patency at one, six, and 12 months was observed in 41 (95%), 29 (67%), and 27 (63%) patients, respectively. Radiocephalic fistulas showed the best performance, with a 12-month patency rate of 100% (4/4), whereas brachiobrachial fistulas showed the lowest rate at 33% (1/3). Statistical analysis demonstrated no significant associations between anatomical site, previous CVC placement, thrombosis risk factors, or previously documented thrombosis and patency at one, six, and 12 months.

Conclusions: Our results highlight the promising results of salvage procedures for AVF in patients undergoing HD, even in a resource-limited setting, demonstrating adequate assisted primary patency duration. More robust prospective studies are needed to validate our findings and to identify prognostic factors associated with long-term AVF patency after endovascular salvage. Additionally, it is critical to develop surveillance protocols focused on fistula research and care, as well as the development of institutional guidelines.

## Introduction

Patients in end-stage kidney disease (ESKD) require renal replacement therapy (RRT), including peritoneal dialysis (PD), hemodialysis (HD), or kidney transplantation. To perform HD, a functional vascular access (VA) is mandatory. The best option is a native arteriovenous fistula (AVFn) rather than an arteriovenous graft (AVG) [1-3]. Compared to AVGs and central venous catheters (CVCs), native AVFs offer significant clinical advantages, including lower rates of infection, thrombosis, and access-related complications [1-3].

However, an arteriovenous fistula (AVF) may fail or suffer complications secondary to mechanical stress, chronic hypertension, intimal hyperplasia, and repeated cannulation [1]. As a consequence, AVF can develop complications such as stenosis, thrombosis, aneurysmal degeneration, or infection, events that may delay HD or require placement of a CVC, which is associated with higher rates of dialytic urgency, catheter-related bloodstream infection, prolonged hospitalization, morbidity, and mortality [3,4]. Among complications, thrombosis is the most frequent cause of AVF failure [5]. 

When these events occur, salvage endovascular interventions such as percutaneous transluminal angioplasty (PTA), thrombectomy, thromboaspiration, or stent placement are indicated to restore fistula patency and ensure the subsequent HD session through the rescued access [1,3]. The primary goal is to address the underlying lesion, preserve the existing fistula, avoid CVC insertion, and reduce healthcare costs [5].

There is substantial variability in the literature regarding the definitions of AVF patency. In this study, we adopted the definitions established by the Spanish Clinical Practice Guidelines on Vascular Access for Hemodialysis. According to this guideline, *primary patency* is defined as the interval between AVF creation and the first access failure or the first required salvage procedure, provided that the fistula had been successfully used for HD before the predefined event [2]. On the other hand, the term *assisted primary patency *refers to the maintenance of patency through interventions performed before access failure occurs; *secondary patency* is defined as the time from creation of the fistula until the permanent abandonment of the access [6]. The main focus of this study is *assisted primary patency* from the first salvage intervention until the next event of failure. 

Because the loss of an AVF leads to decreased venous capital and may necessitate creation of a new access or emergency CVC placement, optimal diagnosis and prompt endovascular management are essential [1,3]. AVF dysfunction carries a substantial economic impact, and the prevalence of ESKD continues to rise in our setting, as do the principal risk factors like diabetes mellitus and hypertension [6].

Given the increasing incidence of ESKD and the growing demand for VA procedures, it is crucial to contribute to the medical literature with detailed descriptions of the demographic and clinical characteristics of patients with AVFns, as well as their patency outcomes after the first salvage intervention. Notably, data on endovascular AVF salvage outcomes from Latin America, and particularly from Colombia, remain scarce. In addition, resource constraints such as delayed administrative approvals, limited access to advanced imaging, and restricted pharmacological availability may uniquely affect access patency in resource-limited settings, underscoring the need for region-specific evidence. The purpose of this study is to describe assisted primary patency at one, six, and 12 months, thereby contributing evidence to support improved outcomes, reduce morbidity and mortality, and ultimately enhance the quality of life of patients undergoing hemodialysis through AVFns.

## Materials and methods

We conducted a retrospective, descriptive, cross-sectional observational study to characterize the population of patients undergoing a salvage procedure of AVFn by the interventional radiology unit and determine which patients had patency of AVFn at one, six, and 12 months. The study was performed at the Hospital Departamental de Villavicencio, a tertiary regional referral center with limited resources located in the Orinoquia region of Colombia, between January 1, 2023, and June 30, 2025. All patients aged 18 years or older who underwent salvage of AVFn for HD were included. Patients were excluded if they had undergone an open surgery salvage procedure, if procedural success could not be determined, or if medical records were incomplete.

Follow-up was conducted retrospectively through electronic medical record review (DINÁMICA software, Advanced Micro Devices, Santa Clara, CA). Patient monitoring was event-driven and based on routine nephrology or dialysis unit visits, with reassessment typically triggered by suspected access dysfunction or inability to perform hemodialysis, reflecting real-world practice in a resource-limited setting. Routine surveillance Doppler ultrasound was not systematically performed. Patency at one, six, and 12 months was determined based on documented clinical usability of the AVFn, defined as the ability to successfully deliver hemodialysis through the salvaged access without need for central venous catheter placement or repeat intervention, as recorded in dialysis reports and clinician notes. Imaging confirmation (Doppler ultrasound or angiography) was used only when clinically indicated. Procedural success was defined as technical restoration of flow with residual stenosis <50% on final angiography, and functional success was characterized by the ability to cannulate the access for hemodialysis.

A non-probabilistic consecutive sampling strategy was applied, including all eligible cases during the study period. Data were collected retrospectively from electronic medical records (DINÁMICA software) and included demographic, clinical, and procedural variables such as age, sex, comorbidities, type of procedure, complications, pharmacological management, AVFn patency, and death. Data were analyzed using RStudio, Version (2024.12.1 + 563). Descriptive statistics were used to summarize data; categorical variables were expressed as frequencies and percentages, while continuous variables were expressed as means or medians. Fisher’s exact test was used for all categorical comparisons due to small sample size and low expected cell counts. Odds ratios are reported only for binary variables. A *P*-value <0.05 was considered statistically significant. Continuous variables were assessed for normality using the Shapiro-Wilk test and compared using the Mann-Whitney U test due to non-normal distribution. The study was approved by the Ethics Committee of the Hospital Departamental de Villavicencio. 

## Results

A total of 43 patients were included in this study group, in which age showed a normal distribution (Shapiro-Wilk test *P *= 0.068), with an average age of 59 years. The study population consisted mostly of men, with 30 patients (70%). 

Baseline characteristics showed hypertension as the most common cause of ESKD leading to hemodialysis in 38 patients (88%) (Table 1). Participants had a mean of two comorbidities, with hypertension being the most frequent (42 patients, 97.7%) (Table 2). A Mahurkar catheter was present in 19 patients (44.18%), and a central venous catheter in 11 patients (26%), indicating that approximately 70% of patients had preexisting VA before salvage. Seven patients (16%) presented with vena cava syndrome at baseline.

**Table 1:** Causes of end-stage kidney disease (N = 43). Values are presented as *n* (%). Percentages were calculated using the total study population (*N* = 43).

Causes of end-stage kidney disease			*n* (%) (*N *= 43 patients)
Hypertension			38 (88.4)
Diabetes mellitus			19 (44.1)
Glomerular disease			3 (7)
Autosomal dominant polycystic kidney disease			1 (2)
Other unspecified causes			14 (32.6)

**Table 2:** Baseline comorbidities (N = 43). Values are presented as *n* (%). Percentages were calculated using the total study population (*N* = 43). *Thrombosis risk factors were defined as uremia, coagulopathy, sepsis, cancer, cirrhosis, and atrial fibrillation.

Baseline comorbidities			*n* (%) (*N *= 43 patients)
Hypertension			42 (97.7)
Diabetes mellitus			19 (44.2)
Coronary artery disease			12(27.9)
Risk factors for thrombotic disease*			10 (23.3)
History of thrombosis			9 (20.9)
Peripheral artery disease			5 (11,6)
Valvular disease			1 (2.3)

**Figure 1:**
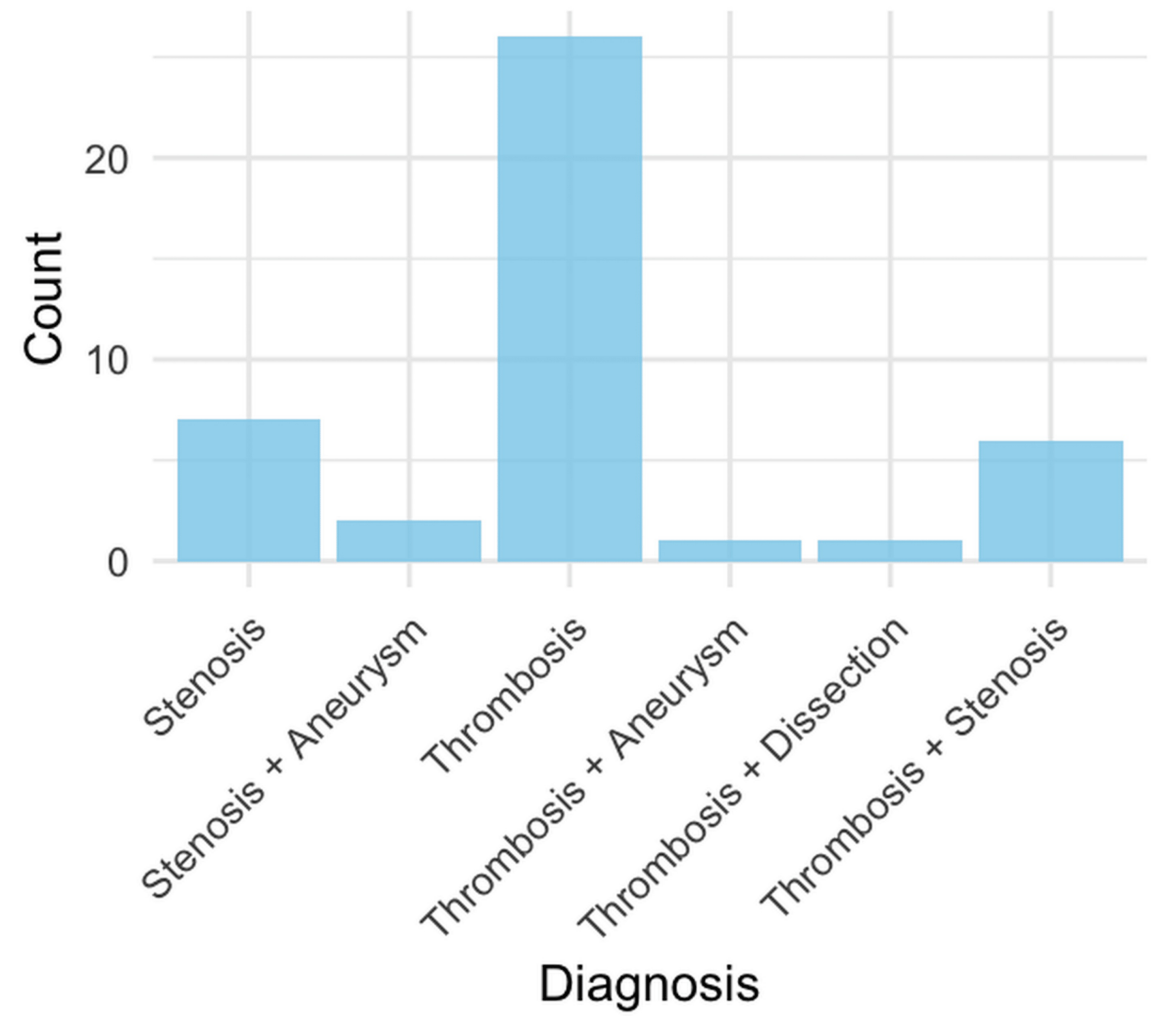
Causes of fistula dysfunction. Bar chart showing the frequency of stenosis, thrombosis, and combined lesions causing arteriovenous fistula dysfunction.

Regarding the AVFs, 26 (60.46%) were left-sided. The most common configuration was brachiocephalic in 32 (74.44%) patients (Table 3). The most common cause of AVF dysfunction included thrombosis in 34 (79.07%) patients, out of which 26 (60%) corresponded only to thrombosis, followed by a combination of thrombosis and stenosis in 6 (14%) cases. Other causes of dysfunction are displayed in Figure 1. 

**Table 3:** Anatomical location and laterality of arteriovenous fistulas (AVFs). Distribution of AVF types according to anatomical location and laterality. Values are presented as absolute frequencies (*N *= 43).

Location	Laterality		Total
	Left	Right	
Radiocephalic	1	3	4
Brachiobasilic	3	1	4
Brachiobrachial	2	1	3
Brachiocephalic	20	12	32
Total	26	17	43

**Figure 2:**
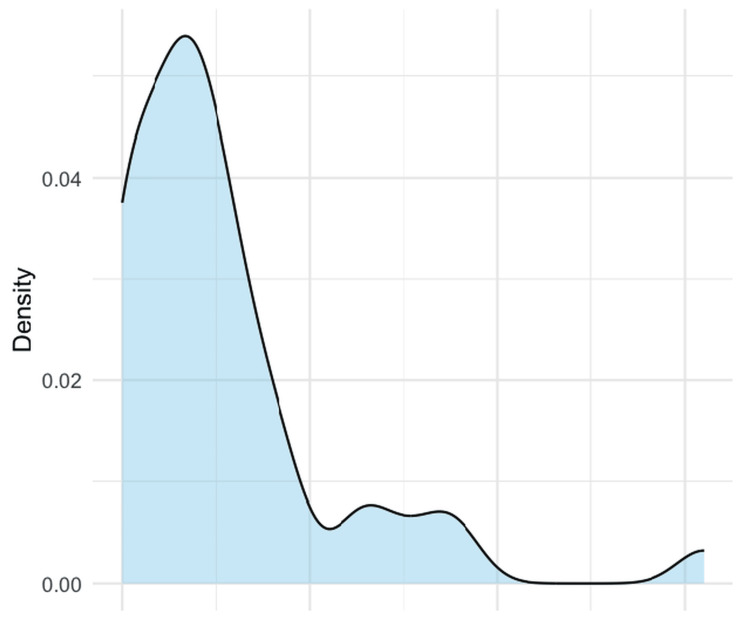
Time to fistula salvage after first dysfunction. Density plot showing the distribution of the time interval (in days) between the first documented fistula dysfunction and the endovascular salvage intervention.

The median time from first documented dysfunction to salvage intervention was 8 days, with 75% of patients treated within 13 days (Figure 2).

In terms of procedures, angioplasty was performed in 42 patients (98%), thromboembolectomy in 22 patients (51%), stent placement in 12 patients (28%), and thromboaspiration in 12 patients (28%). Procedures were not mutually exclusive. Regarding post-procedural pharmacological management, seven patients (16%) received acetylsalicylic acid (ASA), one (2%) received clopidogrel, six (13%) received dual antiaggregation plus anticoagulation, 14 (32.6%) received anticoagulation only, and one patient received a combination of ASA and an anticoagulant. Among anticoagulated patients, apixaban was used in nine, dalteparin in six, and enoxaparin in five. 

Assisted primary patency at one, six, and 12 months after the first salvage procedure was 41/43 (95%), 29/43 (67%), and 27/43 (63%), respectively. Radiocephalic fistulas showed the best performance, with a 12-month patency rate of 4/4 (100%). Brachiocephalic fistulas demonstrated high early patency at one month (29/32, 90.6%), followed by a decline at six months (20/32, 62.5%) and at one year (18/32, 56.25%). Brachiobasilic fistulas remained patent at one month (3/4, 75%) and at one year (2/4, 50%). Lastly, brachiobrachial fistulas had a one-month patency of (2/3, 66.67%), with only (1/3, 33.33%) remaining patent at 12 months, representing the lowest long-term patency among the evaluated configurations.

Statistical analysis 

The number of comorbidities showed a nonnormal distribution (Shapiro-Wilk *W* = 0.833; *P* < 0.001) and did not differ significantly between patients with and without one-month patency (Wilcoxon rank-sum test, *W* = 57.5; *P* = 0.336), six-month patency (Wilcoxon rank-sum test, *W* = 187; *P* = 0.676), or 12-month patency (Wilcoxon rank-sum test, *W* = 210.5; *P* = 0.896). No statistically significant associations were found between patency at 1, 6, or 12 months and anatomical location, thrombosis risk factors, presence of a central venous catheter before repair, Mahurkar catheter use, or history of previous thrombosis (all *P* > 0.05). Detailed statistical results are presented in Tables 4-6.

**Table 4:** Association between categorical variables and one-month AVF patency. This table presents the association between selected categorical clinical and procedural variables and arteriovenous fistula (AVF) patency at one month. Results are reported as odds ratios (ORs) with 95% confidence intervals (95% CIs). Statistical significance was assessed using Fisher's exact test, with *P* < 0.05 considered statistically significant. ^a^Multiple sites refer to involvement of more than one anatomical site, as defined in Table 3. *Thrombosis risk factors were defined as uremia, coagulopathy, sepsis, cancer, cirrhosis, and atrial fibrillation. CI, confidence interval; CVC, central venous catheter; AVF, arteriovenous fistula

Variable	Categories	Odds ratio	95% CI	Statistical test	*P*-value
AVF anatomical location	Multiple sites^a^	N/A	N/A	Fisher’s exact test	0.210
Thrombosis risk factors*	No (33)/Yes (10)	0.29	0.12-3.90	Fisher’s exact test	0.415
CVC before repair	No (32)/Yes (11)	0.00	0.16-4.61	Fisher’s exact test	0.061
Previous Mahurkar catheter placement	No (24)/Yes (19)	∞	0.25-4.85	Fisher’s exact test	0.495
Previous thrombosis	No (34)/Yes (9)	0.25	0.16-7.03	Fisher’s exact test	0.379

**Table 5:** Association between categorical variables and six-month AVF patency. This table presents the association between selected categorical clinical and procedural variables and arteriovenous fistula (AVF) patency at six months. Results are reported as odds ratios (ORs) with 95% confidence intervals (95% CIs). Statistical significance was assessed using Fisher’s exact test, *P* < 0.05. ^a^Multiple sites refer to involvement of more than one anatomical site, as defined in Table 3. *Thrombosis risk factors were defined as uremia, coagulopathy, sepsis, cancer, cirrhosis, and atrial fibrillation. CVC, central venous catheter; AVF, arteriovenous fistula

Variable	Categories	Odds ratio	95% CI	Statistical test	*P*-value
AVF anatomical location	Multiple sites^a^	N/A	N/A	Fisher’s exact test	0.513
Thrombosis risk factors*	No (33)/Yes (10)	0.66	0.12-3.90	Fisher’s exact test	0.704
CVC before repair	No (32)/Yes (11)	0.80	0.16-4.61	Fisher’s exact test	1.000
Previous Mahurkar catheter placement	No (24)/Yes (19)	1.08	0.25-4.85	Fisher’s exact test	1.000
Previous thrombosis	No (34)/Yes (9)	0.96	0.16-7.03	Fisher’s exact test	1.000

**Table 6:** Association between categorical variables and 12-month AVF patency. This table presents the association between selected categorical clinical and procedural variables and arteriovenous fistula (AVF) patency at 12 months. Results are reported as odds ratios (ORs) with 95% confidence intervals (95% CIs). Statistical significance was assessed using Fisher’s exact test, *P* < 0.05. ^a^Multiple sites refer to involvement of more than one anatomical site, as defined in Table 3. *Thrombosis risk factors were defined as uremia, coagulopathy, sepsis, cancer, cirrhosis, and atrial fibrillation. CVC, central venous catheter; AVF, arteriovenous fistula

Variable	Categories	Odds ratio	95% CI	Statistical test	*P*-value
AVF* anatomical location	Multiple sites^a^	N/A	N/A	Fisher’s exact test	0.729
Thrombosis risk factors*	No (33)/Yes (10)	0.86	0.16-5.01	Fisher’s exact test	0.860
CVC before repair	No (32)/Yes (11)	1.05	0.21-5.96	Fisher’s exact test	1.000
Previous Mahurkar catheter placement	No (24)/Yes (19)	1.03	0.25-4.32	Fisher’s exact test	1.028
Previous thrombosis	No (34)/Yes (9)	0.69	0.16-7.03	Fisher’s exact test	0.706

## Discussion

The present study provides a detailed description of the clinical characteristics and assisted primary patency of native AVFs after the first salvage procedure in a regional referral center in the Colombian Orinoquia. Regarding patient characteristics in our cohort the prevalence of comorbidities was comparable to a prior prospective multicenter randomized study, in which common comorbidities included hypertension (97.1%), dyslipidemia (97.1%), type 2 diabetes mellitus (69.6%), coronary artery disease (35%), congestive heart failure (26.8%), and peripheral artery disease (18.9%) [4]. Similar findings were reported in a retrospective Latin American study, in which hypertension was present in 84% of patients, coronary artery disease in 45%, diabetes mellitus in 36%, heart failure in 27%, and peripheral artery disease in 11% [7]. 

These findings highlight the importance of adequate control of chronic non-communicable diseases. In our cohort, hypertension was the most frequent comorbidity, which is expected given its strong association with ESKD [8]. Hypertension may act both as a leading cause of end-stage kidney disease and as a consequence of its underlying pathophysiology, driven by arterial stiffness and enhanced sympathetic activity [8]. In addition, volume overload due to impaired sodium and water excretion contributes to plasma volume expansion and increased cardiac output, further worsening blood pressure control [8].

Most of these patients undergoing hemodialysis have suboptimal blood pressure control. Significant blood pressure fluctuations, particularly intradialytic hypotension and wide variations in systolic blood pressure during hemodialysis, have been associated with a higher risk of AVF thrombosis [9], with intradialytic hypotension reported to nearly double this risk [10].

Regarding fistula dysfunction, the most frequently reported mechanisms are thrombosis and stenosis [11]. In a Latin American study, the incidence of thrombosis and stenosis was variable, ranging from 17 to 25% and from 14 to 42%, respectively [12]. In that report, the incidence of stenosis was higher than that observed in our study [12]. However, our findings are consistent with two Turkish studies, in which thrombosis was more prevalent than stenosis, accounting for 60% of the cases [13,14,15]. Nonetheless, these two conditions may coexist, as thrombosis and stenosis have been reported simultaneously in up to 39.6% of cases [13]. Importantly, thrombosis and stenosis are related pathophysiologically because the proliferation of the venous neointima provokes vascular stenosis, leading to turbulent and reduced flow, which contributes to thrombus formation [12]. 

Our findings demonstrate that endovascular salvage is an effective strategy to recover AVF function, with assisted primary patency rates of 95% at one month, 67% at six months, and 63% at 12 months. An Indian study evaluating balloon angioplasty as the first salvage intervention reported comparable results [16]. In that study, *primary patency* was defined as the time from the first intervention to repeat access thrombosis or repeat intervention, with patency rates of 79% at three months and 60% at six and 12 months, which are consistent with our outcomes [16]. Similarly, a Turkish study using the same definition reported primary patency rates of 72.6%, 69.3%, and 57.5% at three, six, and 12 months for AVFs, respectively [14]. 

Despite these favorable short and mid-term outcomes, long-term patency remains suboptimal. A meta-analysis including 200 studies with 875,269 VAs reported a two-year primary patency of 55% for AVF [15]. This represents a limitation of our study, as we were unable to extend follow-up to 24 months for the study population. 

Regarding anatomical distribution, left-sided fistulas were more common in our cohort, consistent with prior reports [4]. In terms of fistula configuration, radiocephalic fistulas showed the best long-term outcomes with a 12-month patency of 100%, while brachiobrachial fistulas exhibited the lowest 12-month patency of 33.3%. However, these findings should be carefully interpreted due to the wide variability in sample size across anatomical locations. The majority were braquiocephalic (*n* = 32), followed by radiocephalic and braquiobasilic each (*n* = 4), and braquiobraquial (*n *= 3). Importantly, this anatomical distribution varies across the literature and worldwide. For instance, an Indian study reported radiocephalic and brachiocephalic fistulas as the most common configurations, with a lower frequency of brachiobasilic fistulas [16]. 

Direct numerical comparison with previous studies is limited by differences in patency definitions. For example, a Swiss study evaluating 312 AVFs reported primary patency rates at six and 12 months of 39.5% and 34.8%, respectively, for radiocephalic fistulas, with similarly lower rates for brachiocephalic and brachiobasilic configurations [17]. In contrast, our study evaluated assisted primary patency, which incorporates maintenance interventions and therefore represents a distinct endpoint. These differences highlight the heterogeneity of outcome reporting across studies and underscore the influence of local practice patterns, institutional experience, and follow-up strategies on VA outcomes.

A critical factor is the time interval between dysfunction onset and intervention, which has been described as a prognostic factor of assisted primary patency and secondary patency [1,15,18]. In our study, 75% of patients were treated within the first 13 days, with a median of eight days from dysfunction to salvage. The timing of salvage procedures reflects, in part, the way the Colombian healthcare system operates. Specifically, delays frequently depend on administrative authorization processes that are part of the health care model. Some current guidelines, such as the Fístula Code (*Código Fístula*) developed by the Spanish government, recommend urgent intervention within the first 48 hours to reduce hospital stay, decrease the risk of rethrombosis, and improve salvage success rates [1]. Similarly, a study from Singapore implemented a protocol aimed at managing fistula dysfunction within 48 hours; however, even with a structured program, the median time from admission to procedure was 56 hours [15]. Despite this indication, our outcomes were clinically favorable. Notably, one patient underwent successful salvage 63 days after dysfunction and maintained assisted primary patency for almost three years (1,089 days).

Another relevant factor is the pre-existing CVC use before the salvage. In our study, 70% of patients had an alternative VA, reflecting the delay between dysfunction onset and endovascular intervention. This is clinically important, since multiple studies have shown that CVC carries a 4 to 7 times higher risk of infection and a 68% increase in mortality when used as maintenance access [2]. These findings support endovascular salvage as an effective strategy to allow continuity of dialysis while emphasizing the need to establish protocols that accelerate access intervention and improve outcomes in end-stage renal disease.

Furthermore, pharmacologic therapy was heterogeneous in our cohort, with different combinations of anticoagulants and antiplatelet agents administered at the discretion of the treating team. Antiplatelet and anticoagulant regimens were clinician-dependent and heterogeneous, and medication adherence could not be systematically assessed due to the retrospective design. Given the multifactorial nature of VA dysfunction and the broad spectrum of comorbidities in this population, definitive conclusions regarding the impact of pharmacologic strategies on patency outcomes cannot be drawn. Some authors recommend intraprocedural anticoagulation with heparin (50-60 IU/kg as a loading dose, followed by 15-20 IU/kg/hour as maintenance), and post-procedural anticoagulation with intravenous heparin 5000 IU every six hours for at least 24 hours [16]. In other settings, thrombolytic agents such as urokinase are used for thrombus disruption [16]; however, in Colombia, peripheral thrombolysis is currently not permitted, except in selected indications such as acute ischemic stroke. These limitations in pharmacologic options influenced both procedural strategies and post-intervention management in our cohort, reflecting real-world constraints and offering transferable insights for other low-resource settings facing similar challenges.

Additionally, heterogeneity and wide variation in patency definitions and failure criteria in the current literature may lead to misinterpretation and complicate comparisons between studies [17]. Depending on the definition used, reported success rates after AVF interventions may vary substantially [17]. Efforts to standardize terminology and endpoints have been made. In 2018, the Kidney Health Initiative of the American Society of Nephrology published a consensus document proposing standardized definitions and clinical trial endpoints for arteriovenous dialysis access, including *primary patency post-intervention* (time from first intervention to the first event) and *cumulative patency* (time to access abandonment) [19]. For the present study, we used the definition of assisted primary patency, as described in the Spanish Clinical Practice Guidelines on Vascular Access for Hemodialysis [2].

This study has limitations due to its retrospective design. Missing or incomplete records led to the exclusion of some cases, and the overall sample size was relatively small, limiting statistical power to detect significant associations between clinical variables and patency outcomes. Nevertheless, this study provides valuable regional data, as relatively few randomized studies or systematic reviews have addressed this topic, and many previously published cohorts are similarly limited in size.

Finally, additional limitations include the absence of data on length of hospital stay, health system cost outcomes, time to reintervention, or the number of reinterventions before loss of secondary patency. In addition, follow-up duration was relatively short, and longer-term outcomes would be relevant to better characterize access durability.

## Conclusions

Our findings demonstrate that endovascular salvage of nAVFs yields favorable assisted primary patency rates at one, six, and 12 months, even in a resource-constrained setting characterized by administrative delays and limited therapeutic options. These results highlight the value of timely interventional radiology services in preserving VA - the lifeline for patients with ESKD undergoing HD. Preservation of AVFs as permanent VA remains critical in this population, and primary care and nephrology teams play a pivotal role in early recognition of access dysfunction to minimize time-to-intervention and reduce prolonged access compromise.

Future efforts should focus on implementing standardized surveillance protocols, adopting uniform consensus definitions (such as those proposed by the Kidney Health Initiative), and developing institutional *fistula rescue *pathways modeled on successful programs like Spain’s “Código Fístula.” Finally, prospective multicenter studies with longer follow-up are needed to validate these findings, identify prognostic factors associated with long-term patency, and optimize post-intervention management as the global burden of ESRD continues to rise.
